# Safety and tolerability of metformin in overweight and obese patients with dengue: An open-label clinical trial (MeDO)

**DOI:** 10.1371/journal.pntd.0013281

**Published:** 2025-07-01

**Authors:** Nguyet Minh Nguyen, Dong Thi Hoai Tam, Ho Quang Chanh, Huynh Trung Trieu, Nguyen Thi Xuan Chau, Nguyen Thanh Van, Nguyen Thanh Phong, Cao Thi Tam, Nguyen Thi Hong Lan, Luong Thi Hue Tai, Nguyen Thi Cam Huong, Le Thi Ngoc Thuan, Nguyen Van Hao, Phan Tu Qui, Tran Thi Dong Vien, Pham Thi Hai Men, Huynh Thi Thuy Hoa, Phan Vinh Tho, Huynh Thi Le Duyen, Vi Tran Thuy, Thuan Dang Trong, Nguyen Tan Thanh Kieu, Angela McBride, Evelyne Kestelyn, Ronald B. Geskus, Nguyen Lam Vuong, Sophie Yacoub

**Affiliations:** 1 Oxford University Clinical Research Unit, Ho Chi Minh City, Vietnam; 2 Hospital for Tropical Diseases, Ho Chi Minh City, Vietnam; 3 University of Medicine and Pharmacy at Ho Chi Minh city, Ho Chi Minh City, Vietnam; 4 Centre for Tropical Medicine and Global Health, University of Oxford, Oxford, United Kingdom; NIAID DIR: National Institute of Allergy and Infectious Diseases Division of Intramural Research, UNITED STATES OF AMERICA

## Abstract

**Background:**

Despite dengue being a major public health problem, there are no antiviral or adjunctive treatments for the disease. Novel therapeutics are needed, particularly for patients at high risk of severe disease, including those living with obesity. Metformin reduces dengue viral replication in vitro through AMPK activation and may also have beneficial immunomodulatory effects.

**Methods:**

We conducted an open label trial at the Hospital for Tropical Diseases, Ho Chi Minh City, Vietnam, enrolling 120 patients with dengue and obesity (60 treatment arm, 60 control arm receiving standard of care only). Within the treatment arm, the first 10 patients were prescribed low dose metformin, and the remaining 50 patients received weight-based dosing of 1-1.5g/day. The primary outcome was the number of adverse events (AEs), and secondary outcomes were clinical and laboratory parameters, including fever clearance time, platelet nadir, percentage of haematocrit change from baseline, maximum creatinine and highest AST/ALT, and the kinetics of plasma viraemia and NS1 antigenaemia.

**Results:**

The majority of patients in both groups had dengue with warning signs. Six patients in the metformin group and 5 controls developed dengue shock syndrome, and no patients died. There were more AEs recorded in the metformin treated group than in the control group (mean±SD: 15 ± 4 vs. 11 ± 6), particularly the high-dose metformin group (15 ± 5). Twenty-five patients (42%) had to stop the study drug due to AEs, including severe diarrhea (n = 12), dengue shock (n = 5), increased lactate of >3mmol/L (n = 4), hypoglycemia (n = 3), and persistent vomiting (n = 1). There were no clear differences in secondary outcomes between the two groups.

**Conclusions:**

Metformin was poorly tolerated in patients with dengue, mainly due to gastrointestinal side effects. Metformin did not beneficially affect clinical evolution or virological parameters compared to supportive care alone. Our data does not support progression to larger phase 3 trials of metformin in patients with dengue.

**Trial registration:**

ClinicalTrials.gov: NCT04377451 (May 6^th^, 2020).

## Author summary

Dengue is a major public health concern causing millions of infections each year. Among those, people living with obesity are at higher risk of developing severe dengue compared to the general population. There is no effective specific treatment for dengue. Metformin, a glucose lowering drug that has been demonstrated to prevent dengue virus replication (the act of viruses making copies of themselves to increase their number) as well as potentially modulating the patient’s response to dengue virus infection, has been considered as a promising therapy for dengue. In this study, metformin was administered to 60 Vietnamese voluntary patients with dengue and obesity. The number of adverse effects and the clinical progress of dengue disease in the metformin-treated patients were recorded and compared to other 60 Vietnamese patients, who received standard care alone. The study results showed that patients receiving metformin appeared to develop more side effects compared to the standard care controls, leading to 25 patients needed to stop using this drug, mostly due to severe diarrhoea and/or persistent vomiting. In addition, patients receiving metformin did not show improvement in clinical outcomes compared to the standard care group. This study did not support further testing to evaluate the beneficial effects of metformin in larger population with dengue and obesity.

## Background

Dengue is an emerging and neglected vector-borne viral disease, responsible for approximately 100 million symptomatic infections each year [1]. While most people with symptomatic infection have a mild and self-limiting illness, a small proportion progress to develop manifestations of severe dengue, including hypovolemic shock, organ dysfunction, bleeding, and rarely death. Severe illness most frequently occurs during a secondary infection with dengue virus (DENV), when a partially primed but ineffective immune response leads to viral amplification and host-immune driven hyperinflammation [2–4]. There are no licensed antiviral or host-immune modulating therapies for dengue; therapeutics are urgently needed to reduce morbidity and mortality, and lessen the burden on health systems.

Although progression to severe disease is often unpredictable, observational studies have found increased risk among the elderly, pregnant women, and those with metabolic comorbidities, including obesity and diabetes [5]. A systematic review and meta-analysis reported that children with obesity appeared to have significantly higher risk of developing severe dengue in comparison to their counterparts without obesity [6]. Over the past decade, the prevalence of obesity has increased rapidly among children and adolescents in Vietnam, such that 19% and 8% of children between the ages of 5 and 19 years now have overweight and obesity, respectively [7]. In hyperendemic regions, the highest risk of secondary dengue infection typically occurs in late childhood/early adolescence; several countries are now facing confluent dengue and obesity epidemics.

The mechanism underlying enhanced progression to severe disease in patients with obesity is incompletely understood. Potential explanations include (i) chronic low-grade inflammation associated with obesity leads to platelet dysfunction, myocardial injury and endothelial dysfunction, all of which are exacerbated by intercurrent dengue infection; (ii) obesity related downregulation of Adenosine Monophosphate-activated protein kinase (AMPK) leads to an accumulation of lipids in the endoplasmic reticulum, increasing lipid quantities available to form the viral envelope during replication [8,9]; and (iii) altered CD8+ T cell immunity and impairment of natural killer (NK) cell activities in hosts with obesity, leading to higher dengue viraemia [10].

Metformin is an oral anti-hyperglycaemic agent of the biguanide family. Through AMPK activation, it modulates glucose and lipid metabolism, and as a result has been used as a first line drug for type 2 diabetes for 60 years [11,12]. In vitro studies have shown that AMPK activation by metformin also modulates cellular infection with, and replication of the DENV [13], and a retrospective observational study found that diabetes patients taking metformin regularly prior to dengue infection had reduced risk of developing severe dengue versus those not taking metformin [14]. In addition to its proposed antiviral activity, metformin may also have pleomorphic immunomodulatory and/or endothelial stabilising effects, which might be beneficial in DENV infection [15–17].

Metformin is well absorbed, and the time to peak concentration appears to be faster among children with obesity compared to those without [18]. We conducted this trial aiming primarily to access the safety and tolerability of metformin in dengue patients with obesity. In addition, we hypothesized that metformin therapy, given early in the course of dengue disease, would attenuate obesity-induced lipid-inflammatory mediators and improve clinical parameters in patients. We also hypothesized that metformin may reduce viral replication via AMPK activation. Here we report on the safety, tolerability, clinical and virological effects of metformin as an adjunctive therapy for dengue in patients with overweight and obesity.

## Materials and methods

### Ethics statement

Ethical approval for this study was obtained from the Ethics Committee of the Hospital for Tropical Diseases (HTD), the Vietnam Ministry of Health and the Oxford University Tropical Research Ethics Committee.

### Study design and recruitment

We performed an open-label trial of metformin versus standard of care in patients with dengue and overweight or obesity at the HTD in Ho Chi Minh City.

**Fig 1:**
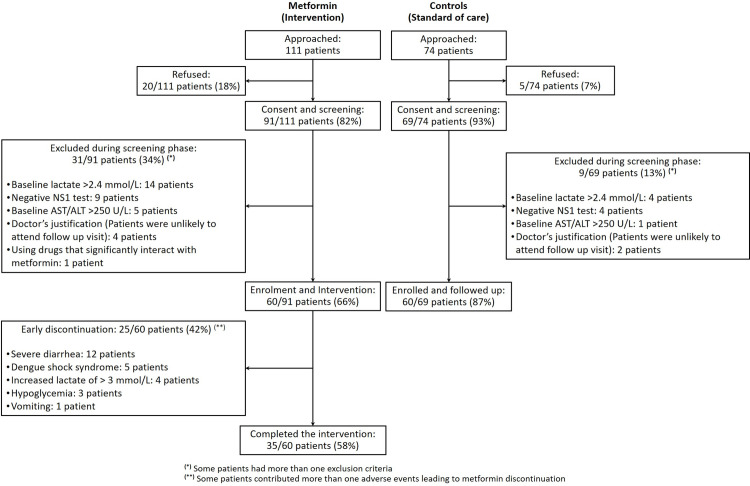
Flowchart of patient recruitment.

The trial was registered on ClinicalTrials.gov (NCT04377451), and the trial protocol has been published elsewhere [19]. Briefly, inpatients aged between 10 and 40 years, admitted to HTD within 72 hours of fever onset, with a clinical diagnosis of dengue and a positive dengue NS1 antigen rapid test, and BMI > 25 kg/m^2^ (in patients aged ≥19 years) or BMI-for-age > 1 standard deviation (SD) above the mean (in those between 10 and 19 years of age) were eligible for recruitment (Fig 1). Exclusion criteria included: localising features suggesting an alternative diagnosis; history of hypersensitivity to metformin; significant diarrhoea and/or vomiting (>3 episodes/24hours); taking metformin or any other regular glucose lowering agents; treatment for heart failure or recent history of myocardial infarction (<12 months); taking any drug with significant interaction with metformin [20,21]; presence of severe dengue at enrolment; baseline parameters: blood glucose <3.9 mmol/L (or <70 mg/dL), alanine transaminase (ALT) and/or aspartate transaminase (AST) >250 U/L, glomerular filtration rate (GFR) <45 mL/min), lactate >2.4 mmol/L.

The patients in this trial were compared to those with overweight/obesity receiving standard of care (no intervention) recruited within a contemporaneously running observational study, with the same inclusion/exclusion criteria. To minimize selection bias, a system was set up on the dengue wards, to ensure eligible patients were recruited into both studies in a systematic way. This involved only one study enrolling for a 2-week time block. During that time, if a patient failed screening assessment or declined enrollment into one of the studies, they were not eligible for the other. All adult patients (≥18 years of age) provided written consent, and in the case of minors (10 – < 18 years old), written assent was provided in addition to the written consent of the parent/guardian.

### Study procedures

Once assigned a study number, patients were provided with a pre-packaged box of metformin, containing sufficient doses for 5 days of treatment plus replacement doses in case of vomiting within 30 minutes of taking the drug. Metformin was administered as directly observed therapy, and patients took the first dose with a light snack as soon as possible after screening. The remaining doses were taken after meals.

In the initial phase (cohort 1), we assessed a low dose of metformin once a day (500 mg in five children aged <16 years, 850 mg in five young adults aged ≥16 years). After satisfactory safety review, we proceeded to assess a higher, weight-based dose regimen of metformin (cohort 2); 1000 mg (500 mg twice daily) for participants with weight <60 kg, and 1500 mg (1000 mg mane, 500 mg nocte) for those with weight ≥60 kg.

Clinical and laboratory assessments were performed daily until hospital discharge or at least 5 days after enrolment to monitor for disease progression and adverse events (AEs). Full blood count (FBC) and point-of-care (POC) lactate tests were carried out daily. Blood glucose levels were measured before each metformin dose. An ultrasound scan was performed on day 5–6 of illness to detect extravascular fluid accumulation (pleural effusion and ascites). Around day 28 of illness, final blood samples were taken for FBC, biochemistry and research assays. Virological (NS1 antigen detection, real time reverse transcription polymerase chain reaction [RT-PCR] assays) and serological tests were performed to confirm the DENV infection, identify infecting serotype and determine patients’ prior immune status (primary versus secondary infection). Daily plasma viraemia levels were quantified using a serotype-specific RT-PCR assay [22].

### Adverse events

AEs were recorded in the case report form and graded using the Common Terminology Criteria for Adverse Events, including both severity and potential causal relationship with the study drug. The AEs were assessed and recorded by the study doctors, including both clinical and laboratory AEs. Serious adverse events (SAEs) were reported to the relevant ethics committees and to an independent data monitoring committee. Further details on the AE reporting can be found in the published protocol [19].

### Data safety monitoring

Safety data for trial participants was reviewed by an independent Data Monitoring Committee (DMC) at predefined intervals according to a detailed plan in the trial protocol [19].

The initial DMC meeting was organised prior to the commencement of the study to review the protocol and agree to a data review schedule and reporting requirements. The second meeting took place after the first ten patients in cohort 1 (low dose) were enrolled. Cohort 2 (higher, weight-based dose) was allowed to start following satisfactory review of all adverse and severe adverse events from cohort 1. Further DMC meetings took place every 6 months to review enrolment, AEs, treatment received, records from follow-up, and any other requested data.

A Trial Steering Committee (TSC) was responsible for providing overall supervision of the conduct of the trial and providing advice as well as the ultimate decision for the continuation of the trial through its independent Chair.

### Pre-defined stopping rules for metformin

Metformin was stopped if patients requested withdrawal from the study, were intolerant of metformin, or if they developed any of the following AEs: moderate-severe acute renal impairment (eGFR < 45mL/min/1.73m^2^), lactate ≥3 mmol/L, AST/AST > 400 U/L, severe diarrhea (≥5 episodes of loose stool/day), persistent vomiting (≥3 episodes/hour or ≥4 episodes/6 hours) and hypoglycaemia (blood glucose <3.9 mmol/L or <70 mg/dL), developed severe dengue (dengue shock syndrome [DSS], severe bleeding, or severe organ impairment including respiratory, cardiac or central nervous system impairment). Where the study drug was stopped due to AEs, participants were followed up until resolution/stabilisation.

### Study outcomes

The primary outcome was to evaluate the safety and tolerability of metformin, measured by the number of AEs occurring in each patient during the five-day treatment course. The secondary outcomes were to evaluate the efficacy of metformin on clinical and virological parameters, which included: fever clearance time (time elapsed from fever onset until the temperature dropped <37.5°C), platelet nadir, percentage haematocrit (HCT) change from baseline (using HCT obtained at the 28-day follow up visit to reflect baseline), highest creatinine and highest AST/ALT measured during the hospitalisation, and the kinetics of plasma NS1 and viremia levels.

### Statistical analysis

All analyses were based on the full analysis set: all patients who received at least one dose of metformin were included in the metformin group, irrespective of stopping the study drug early, and compared with all patients in the control group. All data collected after stopping metformin were also included in the analyses.

The baseline characteristics, evolution of signs and symptoms, AEs, and clinical outcomes were summarized for the two groups using mean and SD or median and 25^th^-75^th^ percentile for continuous variables, and the number of patients and percentage for categorical variables. To test for the differences between groups, we used two-sample t-test for the number of AEs, age, and BMI, Wilcoxon rank-sum test for continuous variables with skewed distribution, and Fisher’s exact test for categorical variables.

The kinetics of laboratory parameters were analysed using linear mixed-effects models for data in the acute phase (within 10 days from symptom onset). In each model, the covariates in the fixed effect included the treatment (metformin versus control), time of measurement from symptom onset (splines with four knots), and the interaction between them. The random effect included an intercept by individual patients and a slope by time with a linear trend. The variables were transformed before the analysis to achieve a relatively normal distribution, except for HCT. The 4th-root transformation was used for platelet count, and log-transformation was used for the other parameters (glucose, lactate, AST, ALT, creatinine, NS1, and viremia). Findings from the models were reported using plots of the predicted mean and 95% confidence interval (CI) of the variables for the two groups.

All analyses were performed with the statistical software R version 4.1.3 [23].

## Results

### Baseline characteristics

Recruitment began in July 2020 and finished in January 2023. There were 111 eligible patients who were approached to participate in the metformin group, of those 91 patients were screened and 60 patients received the metformin intervention. In the control group, 74 patients were approached, of those, 69 patients were screened and 60 patients were recruited (Fig 1).

Baseline patient characteristics are summarized in Table 1. Demographic characteristics (age, sex, BMI, illness day at enrolment) were similar between the two groups. DENV-2 was the predominant infecting serotype in both groups, but the frequency of each serotype differed between the metformin and control groups, with DENV-1 being more frequently observed in the metformin group, and DENV-2 in the non-treatment group. Although the majority of patients in both groups had a secondary infection with dengue, the proportion with primary infection was higher in the metformin group (18.3% in metformin group versus 6.7% in the control group). There was no difference in baseline dengue viral load or quantitative NS1 antigen levels between the two groups. Baseline haematological and biochemical parameters including platelet count, HCT, glucose, lactate, AST, ALT, and creatinine were similar between groups.

**Table 1:** Baseline characteristics.

	Metformin (N = 60)	Control (N = 60)	p-value
Age (years)	18.4 ± 7.9 (10; 37)	17.1 ± 7.1 (10; 37)	0.339
Age group			0.855
Children (<16 years)	32 (53.3)	34 (56.7)	
Adults (≥16 years)	28 (46.7)	26 (43.3)	
Male sex	41 (68.3)	46 (76.7)	0.414
BMI (kg/m^2^)	27.4 ± 3.9 (22; 37)	27.9 ± 4.3 (20; 41)	0.473
Illness day at enrollment			0.083
1	5 (8.3)	0 (0.0)	
2	24 (40.0)	25 (41.7)	
3	31 (51.7)	35 (58.3)	
Serotype			0.070
DENV-1	27 (45.0)	16 (26.7)	
DENV-2	32 (53.3)	42 (70.0)	
DENV-3	0 (0.0)	0 (0.0)	
DENV-4	1 (1.7)	1 (1.7)	
Negative PCR	0 (0.0)	1 (1.7)	
Immune status			0.095
Primary infection	11 (18.3)	4 (6.7)	
Secondary infection	49 (81.7)	56 (93.3)	
Platelet count (K/µl)	160 (101; 207)	156 (115; 194)	0.659
Haematocrit (%)	42 (39; 45)	42 (38; 45)	0.185
Glucose (mg/dl)	110 (93; 134)	116 (102; 127)	0.357
Lactate (mmol/l)	1.6 (1.4; 1.9)	1.5 (1.3; 1.9)	0.175
AST (IU/l)	56 (37; 88)	60 (40; 90)	0.304
ALT (IU/l)	52 (31; 79)	47 (30; 66)	0.998
Creatinine (µmol/l)	76 (63; 87)	73 (60; 86)	0.333
Estimated glomerular filtration rate (ml/min)	135 (117; 150)	147 (127; 165)	0.014
NS1 (log-10 ng/ml)	2.2 (1.7; 2.4)	2.0 (1.8; 2.3)	0.359
Missing data	0	1	
Viremia (log-10 copies/ml)	8.5 (7.6; 9.1)	8.5 (7.7; 9.5)	0.636
Missing data	0	24	

Summary statistics are mean ± SD (range) for age and BMI, median (25^th^; 75^th^ percentiles) for laboratory results, and frequency (%).

AST, aspartate aminotransferase; ALT, alanine aminotransferase; BMI, body mass index; DENV, dengue virus; NS1, non-structural protein 1; PCR, polymerase chain reaction; SD, standard deviation.

### Adverse events, serious adverse events, and discontinuation of metformin

**Fig 2:**
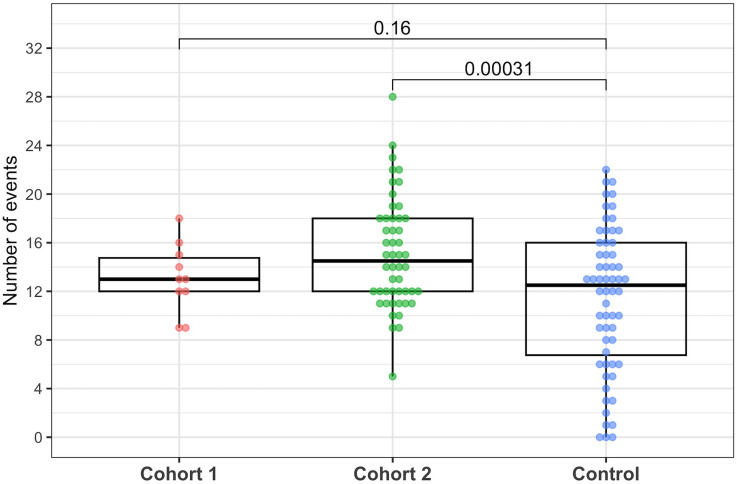
Number of adverse events by treatment group. The line inside each box is the median, the upper and lower margins of each box represent the interquartile range (25^th^ – 75^th^ percentile). Each dot represents the number of AEs of each individual patient, coloured by the treatment groups (cohort 1 contains the first 10 patients with a low dose of Metformin, cohort 2 contains the last 50 patients with a high dose of Metformin, and the control group). The numbers above the plots are the P values comparing each of the two groups (cohort 1 and cohort 2) with the control group using two-sample t-test. AE, adverse event.

The total number of AEs recorded per patient was significantly higher in the metformin group compared to the control group (mean ± SD was 15 ± 4 versus 11 ± 6, respectively, p < 0.001), which was mainly contributed by the high-dose metformin group (15 ± 5 in the high-dose group and 13 ± 3 in the low-dose group) (Fig 2). The majority of AEs were mild (80.2% and 78.5% in the metformin and control groups respectively) and not clinically significant (89.3% and 91% respectively) (S1 and S2 Tables). Six patients in the metformin group and five patients in the control group developed SAEs; these were all attributable to development of DSS.

Metformin was discontinued early in 25/60 (42%) patients: 3/10 patients from the low-dose cohort and 22/50 from the high-dose cohort (S3 Table). Reasons for discontinuation included severe diarrhoea (n = 12), DSS (n = 5), lactate of ≥3 mmol/L (n = 4), hypoglycemia (n = 3), and persistent vomiting (n = 1) (Fig 2).

Table 2 presents a comparison of the predefined AEs for stopping metformin between the two groups (some patients contributed more than one AE to the total). The metformin group had higher proportion of severe diarrhoea (21.7% vs 13.3%), lactate ≥3 mmol/L (15% vs. 8.3%), and hypoglycaemia (10% vs 5%) as compared to the control group. Patients in the metformin group had higher peak lactate levels than those in the control group (2.4 [2.2; 2.7] mmol/L vs 2.1 [1.8; 2.4] mmol/L). Lactate levels peaked late in the disease course for both groups (median 103 and 91 hours respectively) (Table 3). However, patients did not develop any clinical signs or symptoms of lactic acidosis and their lactate levels returned to normal without intervention in all cases. Mild hypoglycemic episodes were recorded in both metformin (10%) and control groups (5%) (Table 2).

**Table 2:** Predefined adverse events for stopping metformin.

	Metformin (N = 60)	Control (N = 60)	p-value
Severe diarrhoea	13 (21.7)	8 (13.3)	0.337
Lactate ≥3 mmol/l	9 (15.0)	4 (6.7)	0.239
Dengue shock syndrome	6 (10.0)	5 (8.3)	1
Hypoglycaemia	6 (10.0)	3 (5.0)	0.491
Persistent vomiting	3 (5.0)	8 (13.3)	0.204
Increased liver enzymes	2 (3.3)	3 (5.0)	1
Severe bleeding	2 (3.3)	0 (0.0)	0.496
Major organ involvement	0 (0.0)	0 (0.0)	
Acute kidney injury	0 (0.0)	0 (0.0)	

Summary statistics are frequency (%).

In the metformin group, 25 patients stopped the drug early. Some patients had more than one predefined adverse event for stopping metformin.

**Table 3:** Clinical outcomes.

	Metformin (N = 60)	Control (N = 60)	p-value
WHO 2009 classification			0.374
Dengue	10 (16.7)	5 (8.3)	
Dengue with warning signs	44 (73.3)	50 (83.3)	
Severe dengue	6 (10.0)	5 (8.3)	
ICU admission	4 (6.7)	4 (6.7)	1
Length of hospital stay (days)	7 (7; 8)	7 (7; 8)	0.491
Missing data	0	1	
Fever clearance time (hours)	101 (82; 119)	108 (88; 127)	0.340
Missing data	1	0	
Platelet nadir (K/µL)	40 (15; 80)	36 (18; 76)	0.755
Highest haematocrit change (%)	12 (8; 18)	13 (9; 17)	0.952
Highest creatinine (µmol/L)	79 (66; 90)	74 (63; 88)	0.308
Highest AST (U/L)	122 (71; 178)	109 (68; 171)	0.652
Highest ALT (U/L)	94 (57; 126)	77 (48; 129)	0.385
Highest lactate level (mmol/l)	2.4 (2.2; 2.7)	2.1 (1.8; 2.4)	<0.001

Summary statistics are median (25^th^; 75^th^ percentiles) and frequency (%)

ALT, alanine aminotransferase; AST, aspartate aminotransferase; ICU, intensive care unit; WHO, World Health Organization.

### Clinical and laboratory outcomes

Table 4 summarises the evolution of warning signs and symptoms during hospitalization. A significantly higher proportion of patients taking metformin reported bruising/petechiae, diarrhoea, and pruritis when compared to controls. There was no significant difference in the proportion of mucosal bleeding between groups. Conversely, extravascular fluid accumulation was observed more frequently in the control versus metformin group (63.3% versus 30.0% respectively).

**Table 4:** Frequency of warning signs and other symptoms during hospitalization.

	Metformin (N = 60)	Control (N = 60)	p-value
Any warning signs	51 (85.0)	55 (91.7)	0.394
- Abdominal pain	40 (66.7)	30 (50.0)	0.095
- Mucosal bleeding	29 (48.3)	25 (41.7)	0.582
- Increase in HCT concurrent with rapid decrease in PLT	27 (45.0)	25 (41.7)	0.854
- Fluid accumulation	18 (30.0)	38 (63.3)	<0.001
- Liver enlargement > 2 cm	15 (25.0)	10 (16.7)	0.369
- Persistent vomiting	5 (8.3)	11 (18.3)	0.178
- Lethargy	1 (1.7)	2 (3.3)	1
- Restlessness	0 (0.0)	0 (0.0)	
Other signs or symptoms	(N = 60)	(N = 57)	
- Bruising/Petechiae	49 (81.7)	31 (54.4)	0.003
- Diarrhoea	47 (78.3)	30 (52.6)	0.004
- Pruritis	39 (65.0)	19 (33.3)	<0.001
- Nausea	38 (63.3)	26 (45.6)	0.065
- Feeling dizzy/faint	30 (50.0)	20 (35.1)	0.135
- Cough	26 (43.3)	14 (24.6)	0.051

Summary statistics are frequency (%).

HCT, haematocrit; PLT, platelet count

Retrospective severity assessment at hospital discharge classified most patients in both groups as having dengue with warning signs (73.3% in the metformin group versus 83.3% in the control group) (Table 3). Eleven patients developed DSS, and there were no deaths. There were no significant differences in length of hospital stay (median 7 days in both groups), fever clearance time, platelet nadir, percentage HCT change from baseline, or peak creatinine, AST, and ALT between groups.

**Fig 3:**
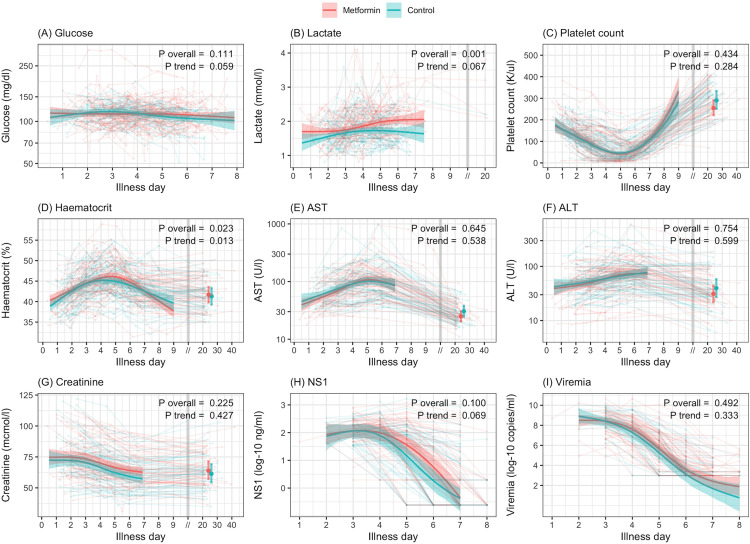
Kinetics of laboratory parameters. Thick-coloured lines and coloured regions represent predicted values and 95% CIs from the linear mixed-effects models. Thick-coloured dots and whiskers represent predicted values and 95% CIs from the linear regression models at the follow-up period after day 18. Light-coloured lines represent the individual trajectory of the parameters. P values are derived from the linear mixed-effects model. P overall represents the overall effect of metformin, and P trend compares the trend over time between the two groups. ALT, alanine aminotransferase; AST, aspartate aminotransferase; CI, confidence interval; NS1, non-structural protein 1.

Fig 3 depicts serial laboratory parameters by illness day, separated by treatment group. There were no significant differences in blood glucose between metformin and control groups, but there was a trend towards higher lactate, evident by day 5–6 of illness in the metformin group. Haematological and biochemistry parameters, including platelet count, AST, ALT and creatinine were similar in both groups. Virological parameters, including daily dengue plasma viremia and quantitative NS1 levels, did not show any significant difference between two groups.

## Discussion

In this manuscript, we have reported the results of an open-label trial assessing the safety and tolerability of adjunctive metformin in patients with overweight and obesity hospitalised with dengue virus infection in Vietnam.

Although overall we did not find a difference in the number of AEs between metformin treatment and control groups, patients in the high-dose metformin group did experience a higher number of AEs versus the control group, predominantly severe diarrhoea. We also found a late increase in lactate levels between days 5 and 7 of illness in the metformin group, which was not seen in the control group. The raised lactate values were observed around the time of clinical recovery and hospital discharge, and as such, the clinical significance of this observation is uncertain.

In addition to these safety signals, we found that metformin was not well tolerated in our study population; 42% of patients in the high dose metformin treatment group stopped the drug prematurely. The most common reason for early discontinuation of metformin was severe diarrhoea and several patients only received 1 or 2 doses of metformin before terminating the study drug due to this adverse effect. Given that we conducted an intention to treat analysis, very early termination may have limited the potential to detect clinical or virological efficacy in the intervention arm.

Metformin commonly causes mild and self-limiting gastrointestinal side effects during the initiation period [21], and for this reason, doses are frequently titrated upwards over several weeks. Such titration is incompatible with the goal of achieving rapid AMPK inhibition during the febrile phase of dengue, hence the patients in this study received high doses from day 1. However, diarrhoea is also a common symptom of acute dengue [24], and although severe diarrhoea was reported by more patients in the metformin treatment group, there was not a statistically significant difference in frequency between the metformin and control groups. Without a randomised and double-blind study design, it is not possible to determine for certain the proportion of diarrhoea directly attributable to metformin treatment.

More patients taking metformin developed mild hypoglycaemia than in the control group. In all cases, this was clinically inapparent and resolved with oral glucose. Hypoglycaemia in patients with dengue has not been widely reported as blood glucose is not routinely measured, despite the common dengue-associated symptoms of anorexia, diarrhoea and vomiting [24]. Taking these observations together, it may be that the side-effect profile of starting high dose metformin do novo is intolerable in addition to the systemic effects of DENV infection. Although concerns regarding tolerability and late lactate increases may limit the application of metformin itself in acute dengue, our ongoing immunopathological studies might still deliver important mechanistic insights regarding the role of AMPK in dengue pathogenesis, potentially highlighting alternative strategies for drug development.

While our results suggest that it may not be appropriate to start metformin de-novo during acute DENV infection, patients on metformin may still benefit from continuing established therapy, providing no contraindications arise. Indeed, a retrospective study from Singapore reported a 33–40% reduction in risk of progression to severe dengue in patients who presented with acute dengue fever already taking metformin, versus those not taking the drug [14]. Although our study was not powered to demonstrate efficacy, our analysis did not suggest that starting metformin during acute dengue had a beneficial effect on dengue viral or NS1 antigen load, and nor did it reduce progression to severe disease, or improve clinical or laboratory parameters. It may be that starting metformin even within 48–72 hours of fever onset is too late to modulate immune and endothelial pathophysiology underlying severe disease, whereas those already established on long-term therapy may benefit from prior induction of protective pathways.

This work represents the first trial of host-directed therapy specifically targeted toward modulating pathophysiology in a patient group with high-risk of progression to severe disease. Although our results do not support the progression of metformin to phase 3 trials, we believe that our targeted approach is a positive step; in a disease where a small minority of patients develop severe complications, patient selection using enrichment criteria such as epidemiological risk factors and/or laboratory parameters is the most viable strategy for targeting host-directed therapeutics to the population most likely to benefit, and reducing side-effect burden among those with a high likelihood of good outcome regardless. Targeted strategies are also more likely to be scalable in high-burden settings.

Further mechanistic studies are ongoing in our group to investigate whether metformin has subclinical immunomodulatory effects, and the impact of metformin on inflammatory and endothelial biomarkers.

Notwithstanding our concerns regarding safety and tolerability of de-novo metformin in dengue, our study does have limitations. This was not a randomised controlled trial, and as such our study may have been subject to sampling bias, by differential patient selection for the treatment arm versus control group based on perceived disease severity or ability to tolerate the study drug. To minimise this risk, we maintained the same selection criteria for entry into both the treatment and control groups and enrolled contemporaneously into both groups to avoid the potential impact of viral serotype variation between dengue seasons. A system was set up on the wards whereby only one study enrolled for a 2-week time block, during that time, if a patient failed screening assessment or declines enrolment into one of the trials, they were not eligible for the other.

In addition, since the study was not blinded, and clinicians working on the trial were necessarily very familiar with the side effect profile of metformin, assessment and recording of clinical outcomes including side effects may also have been subject to observer bias. We sought to minimise this risk by maintaining standardised data collection procedures and ensuring that trial outcomes included objective measurements including laboratory parameters, which would have been independent of observer bias.

## Conclusion

In this cohort of young adults with dengue and overweight or obesity, adjunctive metformin was not well tolerated, and was associated with more severe gastrointestinal symptoms. Metformin did not improve clinical or virological or laboratory parameters associated with dengue severity. The data do not support progression to a larger randomised controlled trial.

## Supporting information

S1 Table Details of adverse events. (DOCX)

S2 Table Specific adverse events by groups. (DOCX)

S3 Table Drug use in Metformin group. (DOCX)

S1 Checklist Consort checklist - CONSORT 2010 checklist of information to include when reporting a randomised trial. (DOCX)

## References

[1] Bhatt S, Gething PW, Brady OJ, Messina JP, Farlow AW, Moyes CL, et al. The global distribution and burden of dengue. Nature. 2013;496(7446):504–7. doi: 10.1038/nature12060 23563266 PMC3651993

[2] Screaton G, Mongkolsapaya J, Yacoub S, Roberts C. New insights into the immunopathology and control of dengue virus infection. Nat Rev Immunol. 2015;15(12):745–59. doi: 10.1038/nri3916 26603900

[3] Srikiatkhachorn A, Mathew A, Rothman AL. Immune-mediated cytokine storm and its role in severe dengue. Semin Immunopathol. 2017;39(5):563–74. doi: 10.1007/s00281-017-0625-1 28401256 PMC5496927

[4] Vuong NL, Le Duyen HT, Lam PK, Tam DTH, Vinh Chau NV, Van Kinh N, et al. C-reactive protein as a potential biomarker for disease progression in dengue: a multi-country observational study. BMC Med. 2020;18(1):35. doi: 10.1186/s12916-020-1496-1 32063229 PMC7025413

[5] Yacoub S, Wills B. Predicting outcome from dengue. BMC Med. 2014;12:147. doi: 10.1186/s12916-014-0147-9 25259615 PMC4154521

[6] Zulkipli MS, Dahlui M, Jamil N, Peramalah D, Wai HVC, Bulgiba A, et al. The association between obesity and dengue severity among pediatric patients: A systematic review and meta-analysis. PLoS Negl Trop Dis. 2018;12(2):e0006263. doi: 10.1371/journal.pntd.0006263 29415036 PMC5819989

[7] Van Minh H, Khuong DQL, Tran TA, Do HP, Watson F, Lobstein T. Childhood Overweight and Obesity in Vietnam: A Landscape Analysis of the Extent and Risk Factors. Inquiry. 2023;60:00469580231154651. doi: 10.1177/00469580231154651

[8] Jeon S-M. Regulation and function of AMPK in physiology and diseases. Exp Mol Med. 2016;48(7):e245. doi: 10.1038/emm.2016.81 27416781 PMC4973318

[9] Soto-Acosta R, Bautista-Carbajal P, Cervantes-Salazar M, Angel-Ambrocio AH, Del Angel RM. DENV up-regulates the HMG-CoA reductase activity through the impairment of AMPK phosphorylation: A potential antiviral target. PLoS Pathog. 2017;13(4):e1006257. 10.1371/journal.ppat.1006257 PMC5383345 28384260

[10] Gallagher P, Chan KR, Rivino L, Yacoub S. The association of obesity and severe dengue: possible pathophysiological mechanisms. J Infect. 2020. 10.1016/j.jinf.2020.04.039 32413364

[11] Rena G, Hardie DG, Pearson ER. The mechanisms of action of metformin. Diabetologia. 2017;60(9):1577–85. doi: 10.1007/s00125-017-4342-z 28776086 PMC5552828

[12] Foretz M, Guigas B, Viollet B. Metformin: update on mechanisms of action and repurposing potential. Nat Rev Endocrinol. 2023;19(8):460–76. doi: 10.1038/s41574-023-00833-4 37130947 PMC10153049

[13] Farfan-Morales CN, Cordero-Rivera CD, Osuna-Ramos JF, Monroy-Munoz IE, De Jesus-Gonzalez LA, Munoz-Medina JE. The antiviral effect of metformin on zika and dengue virus infection. Sci Rep. 2021;11(1):8743. 33888740 10.1038/s41598-021-87707-9 PMC8062493

[14] Htun HL, Yeo TW, Tam CC, Pang J, Leo YS, Lye DC. Metformin Use and Severe Dengue in Diabetic Adults. Sci Rep. 2018;8(1):3344. doi: 10.1038/s41598-018-21612-6 29463812 PMC5820327

[15] Targosz-Korecka M, Malek-Zietek KE, Kloska D, Rajfur Z, Stepien EŁ, Grochot-Przeczek A, et al. Metformin attenuates adhesion between cancer and endothelial cells in chronic hyperglycemia by recovery of the endothelial glycocalyx barrier. Biochim Biophys Acta Gen Subj. 2020;1864(4):129533. doi: 10.1016/j.bbagen.2020.129533 31953127

[16] Isoda K, Young JL, Zirlik A, MacFarlane LA, Tsuboi N, Gerdes N, et al. Metformin inhibits proinflammatory responses and nuclear factor-kappaB in human vascular wall cells. Arterioscler Thromb Vasc Biol. 2006;26(3):611–7. doi: 10.1161/01.ATV.0000201938.78044.75 16385087

[17] Caballero AE, Delgado A, Aguilar-Salinas CA, Herrera AN, Castillo JL, Cabrera T, et al. The differential effects of metformin on markers of endothelial activation and inflammation in subjects with impaired glucose tolerance: a placebo-controlled, randomized clinical trial. J Clin Endocrinol Metab. 2004;89(8):3943–8. doi: 10.1210/jc.2004-0019 15292331

[18] van Rongen A, van der Aa MP, Matic M, van Schaik RHN, Deneer VHM, van der Vorst MM, et al. Increased Metformin Clearance in Overweight and Obese Adolescents: A Pharmacokinetic Substudy of a Randomized Controlled Trial. Paediatr Drugs. 2018;20(4):365–74. doi: 10.1007/s40272-018-0293-1 29748932 PMC6028885

[19] Nguyen NM, Chanh HQ, Tam DTH, Vuong NL, Chau NTX, Chau NVV, et al. Metformin as adjunctive therapy for dengue in overweight and obese patients: a protocol for an open-label clinical trial (MeDO). Wellcome Open Res. 2021;5:160. doi: 10.12688/wellcomeopenres.16053.2 33083561 PMC7539082

[20] Pakkir Maideen NM, Jumale A, Balasubramaniam R. Drug Interactions of Metformin Involving Drug Transporter Proteins. Adv Pharm Bull. 2017;7(4):501–5. doi: 10.15171/apb.2017.062 29399540 PMC5788205

[21] NHS. Metformin. 2022 [updated 24 March 2022; accessed date: 15 April 2024]. https://www.nhs.uk/medicines/metformin/

[22] Hue KDT, Tuan TV, Thi HTN, Bich CTN, Anh HHL, Wills BA, et al. Validation of an internally controlled one-step real-time multiplex RT-PCR assay for the detection and quantitation of dengue virus RNA in plasma. J Virol Methods. 2011;177(2):168–73. doi: 10.1016/j.jviromet.2011.08.002 21843553 PMC4347661

[23] R Core Team. R: A Language and Environment for Statistical Computing. 2023.

[24] World Health Organization. Dengue: Guidelines for Diagnosis, Treatment, Prevention and Control: New Edition. Geneva: World Health Organization; 2009. 23762963

