# Supplementary material for: Safety and tolerability of metformin in overweight and obese patients with dengue: An open-label clinical trial (MeDO)
Source: PLoS Negl Trop Dis. 2025 Jul 1;19(7):e0013281. doi: 10.1371/journal.pntd.0013281 (PMC12237267; doi:10.1371/journal.pntd.0013281)
Supplement: S1 Table — (DOCX) [file pntd.0013281.s001.docx]

### S1 Table. Details of adverse events

|  | Cohort 1 | Cohort 2 | Metformin | Control |
| --- | --- | --- | --- | --- |
| Number of AEs recorded | 131 | 755 | 886 | 683 |
| Type of AE |  |  |  |  |
| Laboratory | 82 (62.6) | 465 (61.6) | 547 (61.7) | 453 (66.3) |
| Clinical | 49 (37.4) | 290 (38.4) | 339 (38.3) | 230 (33.7) |
| Relation to study drug |  |  |  |  |
| Related to study drug | 0 (0.0) | 0 (0.0) | 0 (0.0) | 0 (0.0) |
| Probably related to study drug | 0 (0.0) | 7 (0.9) | 7 (0.8) | 0 (0.0) |
| Possibly related to study drug | 16 (12.2) | 36 (4.8) | 52 (5.9) | 0 (0.0) |
| Unlikely to be related to study drug | 6 (4.6) | 157 (20.8) | 163 (18.4) | 0 (0.0) |
| Not related to study drug | 109 (83.2) | 555 (73.5) | 664 (74.9) | 36 (100.0) |
| CTCAE Grade |  |  |  |  |
| Grade 1 | 97 (74.0) | 603 (79.9) | 700 (79.0) | 530 (77.9) |
| Grade 2 | 25 (19.1) | 115 (15.2) | 140 (15.8) | 109 (16.0) |
| Grade 3 | 5 (3.8) | 28 (3.7) | 33 (3.7) | 36 (5.3) |
| Grade 4 | 4 (3.1) | 9 (1.2) | 13 (1.5) | 5 (0.7) |
| Grade 5 | 0 | 0 | 0 | 0 |
| Missing data | 0 | 0 | 0 | 3 |
| Clinically significant (yes) | 24 (18.3) | 71 (9.4) | 95 (10.7) | 60 (9.0) |
| Missing data | 0 | 0 | 0 | 17 |
| Required specific treatment | 21 (16.0) | 89 (11.8) | 110 (12.4) | 70 (10.2) |

Summary statistics are frequency (%). The percentages are calculated from the number of AEs for each column. Cohort 1 contains the first 10 cases with a low dose of metformin. Cohort 2 contains the last 50 cases with a high dose of metformin.

AE, adverse event; CTCAE: Common Terminology Criteria for Adverse Events
