# Supplementary material for: Safety and tolerability of metformin in overweight and obese patients with dengue: An open-label clinical trial (MeDO)
Source: PLoS Negl Trop Dis. 2025 Jul 1;19(7):e0013281. doi: 10.1371/journal.pntd.0013281 (PMC12237267; doi:10.1371/journal.pntd.0013281)
Supplement: S2 Table — (DOCX) [file pntd.0013281.s002.docx]

### S2 Table. Specific adverse events by groups

|  | Cohort 1 (N=10) | Cohort 2 (N=50) | Metformin (N=60) | Control (N=60) |
| --- | --- | --- | --- | --- |
| Decreased platelet count | 8 (80.0) | 42 (84.0) | 50 (83.3) | 42 (70.0) |
| Decreased neutrophil count | 8 (80.0) | 39 (78.0) | 47 (78.3) | 37 (61.7) |
| Hepatomegaly | 8 (80.0) | 34 (68.0) | 42 (70.0) | 31 (51.7) |
| Itching/Rash | 4 (40.0) | 38 (76.0) | 42 (70.0) | 28 (46.7) |
| Diarrhoea | 5 (50.0) | 37 (74.0) | 42 (70.0) | 25 (41.7) |
| Thickened gallbladder wall | 5 (50.0) | 34 (68.0) | 39 (65.0) | 36 (60.0) |
| Abdominal pain | 9 (90.0) | 25 (50.0) | 34 (56.7) | 19 (31.7) |
| Increased liver enzyme | 4 (40.0) | 29 (58.0) | 33 (55.0) | 34 (56.7) |
| Increased HCT/HGB | 3 (30.0) | 29 (58.0) | 32 (53.3) | 29 (48.3) |
| Decreased white cell count | 6 (60.0) | 26 (52.0) | 32 (53.3) | 26 (43.3) |
| Skin bleeding | 3 (30.0) | 29 (58.0) | 32 (53.3) | 22 (36.7) |
| Increased lactate | 2 (20.0) | 20 (40.0) | 22 (36.7) | 12 (20.0) |
| Mucosal bleeding | 3 (30.0) | 18 (36.0) | 21 (35.0) | 18 (30.0) |
| Vomiting | 4 (40.0) | 17 (34.0) | 21 (35.0) | 10 (16.7) |
| Nausea | 4 (40.0) | 17 (34.0) | 21 (35.0) | 10 (16.7) |
| Pleural effusion | 4 (40.0) | 13 (26.0) | 17 (28.3) | 27 (45.0) |
| Decreased lymphocyte count | 3 (30.0) | 13 (26.0) | 16 (26.7) | 21 (35.0) |
| Decreased albumin | 4 (40.0) | 9 (18.0) | 13 (21.7) | 12 (20.0) |
| Ascites | 2 (20.0) | 9 (18.0) | 11 (18.3) | 8 (13.3) |
| Dizziness | 2 (20.0) | 9 (18.0) | 11 (18.3) | 7 (11.7) |
| Dengue shock syndrome | 1 (10.0) | 5 (10.0) | 6 (10.0) | 5 (8.3) |

Summary statistics are frequency (%). Cohort 1 contains the first 10 cases with a low dose of metformin. Cohort 2 contains the last 50 cases with a high dose of metformin. This table shows the most common adverse events reported by the investigators (10% or above).

APTT, activated partial thromboplastin; HCT, haematocrit; HGB, haemoglobin; PT, prothrombin time
