# Supplementary material for: Safety and tolerability of metformin in overweight and obese patients with dengue: An open-label clinical trial (MeDO)
Source: PLoS Negl Trop Dis. 2025 Jul 1;19(7):e0013281. doi: 10.1371/journal.pntd.0013281 (PMC12237267; doi:10.1371/journal.pntd.0013281)
Supplement: S3 Table — (DOCX) [file pntd.0013281.s003.docx]

### S3 Table. Doses of metformin received

|  | All patients  (N=60) | Cohort 1  (N=10) | Cohort 2  (N=50) |
| --- | --- | --- | --- |
| Total doses of metformin received ^a^ |  |  |  |
| 1 | 7 (11.7) | 2 (20.0) | 5 (10.0) |
| 2 | 8 (13.3) | 0 (0.0) | 8 (16.0) |
| 3 | 1 (1.7) | 0 (0.0) | 1 (2.0) |
| 4 | 4 (6.7) | 1 (10.0) | 3 (6.0) |
| 5 | 8 (13.3) | 7 (70.0) | 1 (2.0) |
| 9 | 4 (6.7) | 0 (0.0) | 4 (8.0) |
| 10 | 28 (46.7) | 0 (0.0) | 28 (56.0) |
| Early discontinuation of metformin | 25 (41.7) | 3 (30.0) | 22 (44.0) |

Summary statistics are frequency (%).

^a^ The complete treatment included 5 doses of metformin in cohort 1, and 10 doses of metformin in cohort 2.
